# Functions of Plexins/Neuropilins and Their Ligands during Hippocampal Development and Neurodegeneration

**DOI:** 10.3390/cells8030206

**Published:** 2019-02-28

**Authors:** Vanessa Gil, José Antonio del Río

**Affiliations:** 1 Molecular and Cellular Neurobiotechnology, Institute for Bioengineering of Catalonia (IBEC), The Barcelona Institute of Science and Technology (BIST), Parc Científic de Barcelona, 08028 Barcelona, Spain; 2 Department of Cell Biology, Physiology and Immunology, Universitat de Barcelona, 08028 Barcelona, Spain; 3 Center for Networked Biomedical Research on Neurodegenerative Diseases (CIBERNED), 08028 Barcelona, Spain; 4 Institute of Neuroscience, University of Barcelona, 08028 Barcelona, Spain

**Keywords:** plexinD1, sema3E, neuropilins, neuronal migration, synapse formation

## Abstract

There is emerging evidence that molecules, receptors, and signaling mechanisms involved in vascular development also play crucial roles during the development of the nervous system. Among others, specific semaphorins and their receptors (neuropilins and plexins) have, in recent years, attracted the attention of researchers due to their pleiotropy of functions. Their functions, mainly associated with control of the cellular cytoskeleton, include control of cell migration, cell morphology, and synapse remodeling. Here, we will focus on their roles in the hippocampal formation that plays a crucial role in memory and learning as it is a prime target during neurodegeneration.

## 1. Introduction

Cell migration and axonal navigation play essential roles in tissue and organ formation during neural development. In a broad sense, the cerebral cortex is one of the most intricate regions of the mammalian brain, and both complex cell migration and axonal wiring processes are required to generate the high level of organization observed in this brain area [1]. Defects in neuronal migration or axonal navigation during their development may lead to severe learning and cognitive deficits such as autism, lissencephaly, epilepsy, and schizophrenia, among others [1,2,3]. Thus, understanding signals controlling cell migration and/or axon navigation is essential to discriminate the mechanisms underlying normal development and pathological alterations. To date, the full repertoire of molecular factors implicated in these processes has not been completely revealed. However, it is evident that the putative underlying mechanisms involve different classes of signaling molecules, and the list of potential candidates for the modulation of growth cone dynamics and neuronal migration is continuously expanding [4]. Another relevant point is that for several molecules, the expression and function of their receptors is under well-established molecular control. Such control leads to establishing a dynamic spatiotemporal regulation of the responsiveness of growth cones and navigating neurons to specific guidance cues. In fact, axons travel spatially and temporally across several regions of the developing brain that contain a variety of guidance molecules and factors. In addition, many molecules define anatomical boundaries by being expressed in several areas and producing different responses depending on the receptor complexes expressed by projecting neurons as a function of time and space [5]. In addition, during aging and in certain pathological conditions (i.e., trauma and axotomy), dormant cues with key roles during development are reactivated and overexpressed in particular reactive cells, with negative results in most cases [6]. In this review, we will focus anatomically on one of the “oldest” regions, in evolutionary terms, of the mammalian cerebral cortex: the hippocampal formation.

## 2. The Hippocampal Formation: A Three-Layered Allocortex Model for Axon Development and Regeneration

**Figure 1:**
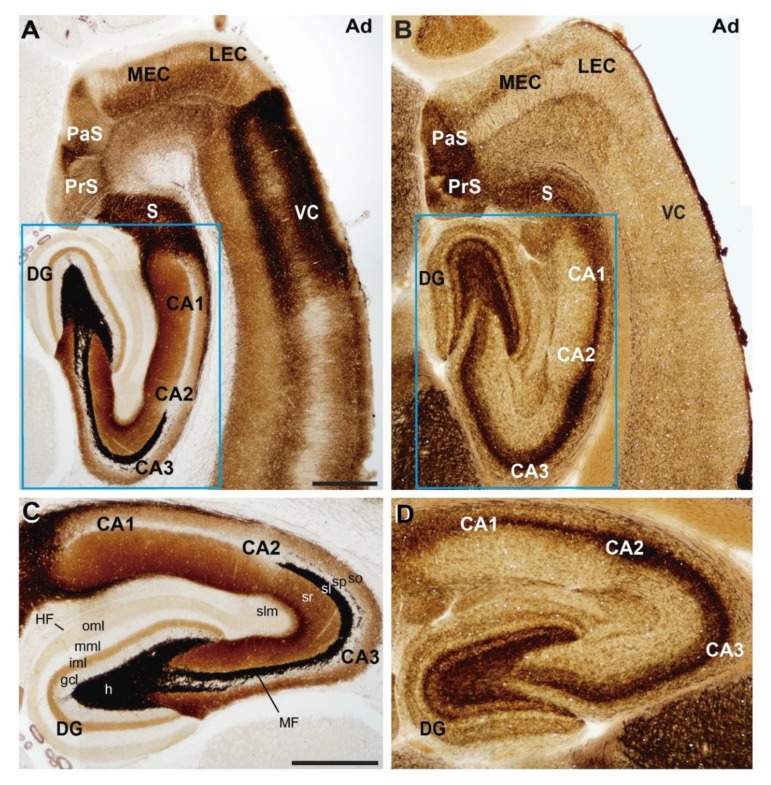
Anatomical organization of the rat hippocampal formation. Photomicrographs illustrating the pattern of (**A**,**C**) selenite-silver staining (TIMM) and (**B**,**D**) acetylcholinesterase reaction in horizontal sections of adult rats. Both stainings illustrate the distribution of the main regions and layers of the hippocampal formation. A higher magnification of the blue boxed area in (**A**,**B**) is shown in (**C**,**D**) respectively. Abbreviations: CA1–3 = cornus ammonis region 1–3; DG = dentate gyrus; gcl = granule cell layer; h = hilus; HF = hippocampal fissure; iml = inner molecular layer; LEC = lateral entorhinal cortex; MEC = medial entorhinal cortex; MF = mossy fiber bundle; mml = medial molecular layer; oml = outer molecular layer; PaS = parasubiculum; PrS = presubiculum; S = subiculum; sl = stratum lucidum; slm = stratum lacunosum-moleculare; so = stratum oriens; sp = stratum pyramidale; sr = stratum radiatum; VC = ventrolateral neocortex. Scale bars: A = 500 μm pertains to (B); C = 500 μm pertains to (D).

The mammalian cerebral cortex can be subdivided into the neocortex, the olfactory cortex, and the hippocampal region (HR). The allocortex and the HR are a three-layered structure in comparison to the six-layered structure observed in the mammalian neocortex (see [7] for review). In addition, the HR contains what was formerly called the “periallocortical region”, which is made up of the presubiculum, the area retrosplenialis, the parasubiculum, and the entorhinal region. In between the allocortex and periallocortex, we find the hippocampal formation formed by the fascia dentata or dentate gyrus, the hippocampus proper, and the subiculum [8,9] (Figure 1).

**Figure 2:**
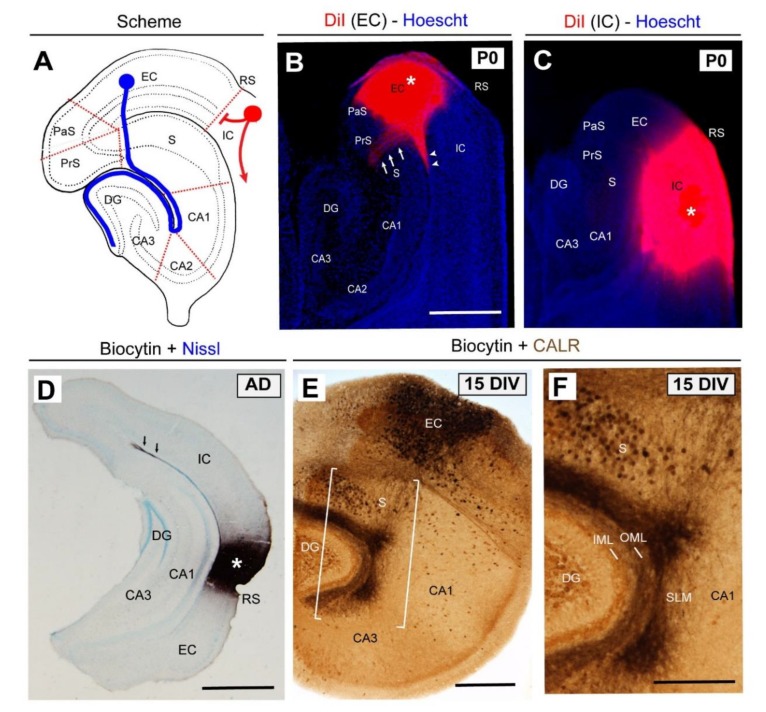
Lamina-specific innervation of the entorhino-hippocampal (EH) pathway. (**A**) Scheme of the hippocampal formation illustrating the EH (blue) and corticofugal (red) connections. (**B**) Pattern of EH innervation after DiI injection in the entorhinal cortex (asterisk) at P0. Note that entorhinal axons cross the subiculum (arrows) to reach the hippocampus proper. In parallel, entorhinal axons are also able to reach the CA1 of the hippocampus proper through the alveolar pathway (arrowheads). (**C**) In contrast, when DiI is injected in the isocortical region, no labeled axons are detected in the hippocampus. (**D**) Photomicrograph illustrating the lack of staining in the hippocampus when byocitin is injected in the perirhinal cortex (asterisk) in vivo in an adult animal. (**E**) Pattern of EH regeneration in organotypic slices after 15 DIV. (**F**) High-magnification of the boxed area in (E). Abbreviations as in Figure 1 and EC = entorhinal cortex; CALR = calretinin immunostaining; DIV = days in vitro; IC = isocortex, and RS = rhinal sulcus. Scale bars: B = 500 μm pertains to (C); D = 500 μm; E = 250 μm and F = 100 μm.

The hippocampal formation plays crucial roles in the consolidation of information from short- to long-term memory, as well as in spatial memory [10,11]. Since principal neurons, the main extrinsic afferent connections (i.e., entorhinal or commissural/associational fibers) and the most relevant intrinsic connection (i.e., the mossy fibers) are organized into well-defined lamina in the hippocampus [12], the hippocampal formation is an important model to help in understanding the mechanisms controlling axon pathfinding and axon regeneration. During axonal wiring in perinatal development in rodents, axons from the neocortical regions close to the entorhinal cortex (ventrolateral neocortex) mainly avoid the hippocampal region (including the hippocampal and retrohippocampal formation [13]). In addition, entorhinal axons avoid invading the adjacent ventrolateral isocortex, entering the hippocampal formation to reach the stratum lacunosum moleculare (SLM) of the hippocampus proper to further innervate the outermost portion of the molecular layer (OML) of the dentate gyrus [8,13,14,15] (Figure 2A). In fact, based on classical neuroanatomical studies using axonal tracers (e.g., [13,14,15]), cell transplantation in vivo (e.g., [16,17,18]) and in vitro in slices [19,20,21,22,23,24], it has been reported that axons from entorhinal neurons are able to specifically reach the SLM/OML of the hippocampal formation in healthy conditions. This lamina-specificity distribution of entorhinal axons in the hippocampus can be visualized by injecting in vivo an axonal tracer such as byocitin or DiI ((1,1′-dioctadecyl-3,3,3′3′-tetramethylindocarbocyanine perchlorate) into the entorhinal cortex (Figure 2A–D). This is also maintained in entorhino-hippocampal slices in vitro, where the entorhinal projecting axons are axotomized during the co-culture preparation. An example of the specific development of the connection can be seen in Figure 2D,E (but see also refs [18,20,25,26] for additional examples). One interesting feature of the entorhino-hippocampal connection also lies in the fact that entorhinal axons cross the subiculum to reach the hippocampus proper. This anatomical distribution has led researchers to describe the entorhino-hippocampal connections as a “perforant” pathway [14].

In addition, axons forming the second main afferent connection of the hippocampus proper, the commissural/associational pathway, avoid the SLM/OML in vivo [13,14,15,27] as well as in vitro [23,25,26]; they are restricted to the stratum radiatum of the hippocampus proper and the innermost portion of the molecular layer (IML) of the dentate gyrus [8]. Only after lesion of the entorhinal fibers are the commissural/associational axons able to reinnervate the SLM/OML [25]. With respect to mossy fibers, postnatal and adult newborn granule cells extend their axons, forming synaptic contacts on hilar mossy cells and on proximal dendrites of the CA3 pyramidal cells in the stratum lucidum [8,12]. 

**Figure 3:**
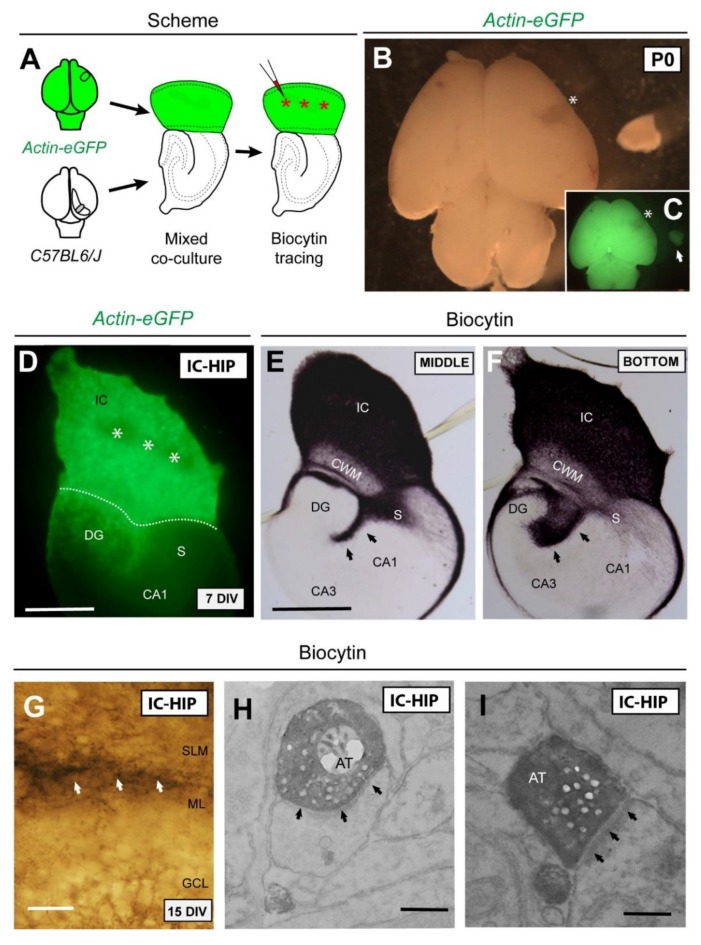
Schematic diagram illustrating the experimental design. (**A**) A small piece of the isocortex (parietal cortex) from Actin-eGFP mice at P0 was transplanted in replacement of the original entorhinal cortex of C57BL6/J in order to perform a mixed iso-hippocampal co-culture. After 6 DIV, biocytin tracer was injected in the isocortical region (asterisks). (**B**,**C**) Bright-field and fluorescence micrographs of Actin-eGFP brain showing the dissected area (asterisk). (**D**) After 7DIV, eGFP-positive isocortical axons were seen crossing the subiculum and entering the hippocampus. Dashed line indicates the boundary between the isocortex and hippocampus. (**E**,**F**) Sections from the middle and the bottom of the organotypic co-culture illustrating biocytin-labeled axons from the isocortical region. (**G**) A high-magnification of biocytin-labeled axons (arrows) innervating the SLM/ML boundary. (**H**,**I**) Electron micrographs showing asymmetric contacts (arrows) established between isocortical axons and apical dendrites of granular neurons. Abbreviations as in Figure 1 and Figure 2 and AT = axonal terminal; CWM = cortical white matter. Scale bars: D = 250 μm; E = 250 μm pertains to (F); G = 50 μm; H = 1 μm pertains to (I).

These fascinating features of axonal wiring and connections invite researchers to examine in depth this characteristic regional distribution of neocortical vs. allocortical cortical axons as well as the intrinsic laminated termination of hippocampal afferents. Although different evolutionary-related issues should be considered [8], we need to focus on the specific neuronal information processing that takes place in the entorhinal cortex and the hippocampus. This information is largely related to the processing of the spatial information in broad (entorhinal cortex) or more specific (hippocampus) locations due to the roles of “grid cells” in the medial entorhinal region and “place cells” in the hippocampus proper [28]. As a whole, the mammalian cerebral cortex requires a high degree of functional specialization among the regions, but more relevantly there is a need to establish and preserve this regionalization from the very beginning during development. In fact, from an anatomical point of view, the allocortex fails to cleave completely from the mantle layer, while the neocortex completely separates from the mantle layer at the beginning of neural development [8]. Thus, in order to obtain this high degree of cortical specialization, different developmental and epigenetic processes need to be developed. Molecular biology studies will help reveal what is responsible for this functional regionalization of the cerebral cortex. Indeed, as in a high level “computer”, subtle wiring is needed to achieve appropriate functional homeostasis and information processing of the system. Thus, “molecular barriers” are established between these “three-layered” cortices and the “six-layered” ones during evolution. However, we can “steal” years from natural evolution; if we confront slices of the parietal cortex and hippocampus in the absence of additional allocortical regions, we are able to establish an “aberrant” connection between the parietal cortex and the hippocampus that is distributed similarly to the entorhino-hippocampal connection, with asymmetric contacts between neocortical neurons and the apical dendritic regions of hippocampal or granule cell neurons (after biocytin staining of parietal axons) (see Figure 3). These “errors” in target selection in vitro were also described by Molnár and Blakemore when they cultured thalamic regions with isocortex [29]. This reinforces the notion of the existence of these molecular “barriers” between different cortical regions. However, these observations also suggest that we must take into account in our research not only the source and target of a particular connection, but also the spatio-temporal patterns of the axonal guidance molecules and their receptors involved in the definition of large territories as well as those that define cell-axon recognition and connectivity at a cellular level.

One of the first molecular barriers described in the cortical regions, forcing entorhinal axons to navigate towards the hippocampus and avoid other neocortical areas, was Sema3F [30]. It is noteworthy that the final target also plays an important role in the correct establishment of this connection [27,31]. Therefore, it seems likely that axonal pathfinding and fiber segregation into well-defined lamina in the hippocampal formation is a well-orchestrated process that depends on multiple factors such as the involvement of long-range cues influencing early axonal trajectories, membrane- or substrate-anchored cues providing layer-specific positional information along the pathway, and “chemotrophic” attraction cues or stop signals from the target area [32,33,34]. Research in this field during the last three decades has provided a large number of molecules implicated in axonal guidance processes, and four major families have been identified: the slits, ephrins, netrins, and semaphorins, as well as some morphogens such as Wnts, SHH, and BMPs [35,36,37]. Members of all of these families have been implicated in hippocampal axonal specification [30,38,39,40] as we discuss later. Among these, great interest has been focused on the class 3 secreted semaphorins family; and different members and/or combinations of members have been described as playing roles in different hippocampal connections and their refinements.

## 3. Semaphorins and Their Receptors

The semaphorin superfamily comprises three families of proteins on the basis of structural criteria: semaphorins, plexins and the MET and RON receptor tyrosine kinases (RTKs). The distinctive structural and functional element of these families is the sema domain, an approximately 500-amino acid extracellular domain important for dimerization and interaction specificity [41]. Semaphorins are a large and diverse family of axon guidance molecules with key roles in the peripheral and central nervous system [42]. This family contains more than twenty members grouped into eight classes, based on sequence similarities and their structural forms [43]. Apart from the sema domain, semaphorins contain a plexin–semaphorins–integrin (PSI) domain and a C-terminus domain, which confer class-specific features [44]. Both the sema and PSI domains are also present in plexins and MET and RON tyrosine kinases. Classes 1 and 2, and also one member of class 5 (Sema5C), are expressed in invertebrates, while class V semaphorins are virally encoded. Vertebrates express the remaining classes, namely, class 3 semaphorins (A–G) which are secreted, and classes 4 to 7 (4A to D, F and G, 5A and B, 6A to D and 7A) which are all membrane-bound, although proteolytic cleavage resulting in the secretion of some members (e.g., Sema5B and Sema4A) has been reported [45,46].

Semaphorins (classes 4 to 7) act as ligands whose principal receptors are plexins, which are signal-transducing subunits that are divided into four classes (A1–4, B1–3, C1, and D1) [47]. The plexin extracellular region contains several different motifs and domains, including a divergent sema domain, whereas the intracellular region always contains a GTPase-activating protein (GAP) domain (reviewed in [48]). However, other membrane-associated semaphorins can also act as receptors or co-receptors for other semaphorins, a phenomenon known as reverse signaling. In contrast, class 3 semaphorins require neuropilins as co-receptors (ligand-binding subunits) with the plexins [49]. Neuropilins are type I transmembrane proteins that harbor a short cytoplasmic domain, and to date, two members have been identified in vertebrates: Neuropilin 1 and 2 (Np1 and Np2, respectively). Therefore, neuropilins act as ligand-binding subunits and plexins are signaling subunits of class 3 semaphorin holoreceptor complexes. Exceptions to this are Sema3E which binds directly to PlexinD1 [50], and Sema3C which can also act through PlexinD1 in the absence of neuropilins [51]. In addition, some semaphorins exert their function by binding to various receptor complexes associated with plexins, such as tyrosine kinase receptors MET [52], ErbB2 [53], and VEGFR2 [54], but also extracellular matrix receptors such as alpha and beta integrins [55] and proteoglycans ([56] for review).

The earliest described role for semaphorins was in axon guidance in the development of both the central [30,57] and peripheral nervous system ([58,59,60] and recently reviewed in [61]; however, they have been implicated in other completely different biological functions such as vascular morphogenesis [62,63], neural crest cell migration [64,65], tumor progression [66,67], immune system functioning [68,69], homeostasis of hormone systems [5,70], and bone development and remodeling [71]. In the nervous system, their best described functions are in axon tract and synapse formation through cytoskeleton reorganization of the axonal growth cone [72], although an independent signaling mechanism has been described for Sema3A [73]. Other roles reported are neuronal apoptosis [74], dendrite growth [75,76,77,78], neuronal migration [79,80], and spine formation in the dorsal root ganglion and brain [81]. Although most of these functions are exclusively related to neuronal features, it must be mentioned that plexin/semaphorin signaling also mediates communication between glia and motoneurons, which is essential for the formation of functional myotopic maps [82].

## 4. Semaphorins, Their Receptors, and Hippocampal Development

As mentioned above, the hippocampal formation presents a well-organized architecture: principal cells are located in single layers and afferents (entorhinal and commissural/associational axons) and intrinsic connections are distributed in a layer-specific termination [32,33,34]. Members of all four families of guidance factors have been involved in hippocampal axonal specification [38,39,40] with class 3 semaphorins being the most studied so far. The list of receptors and co-receptors that participate in this Sema3s-mediated signaling is continuously growing, and a great variety of combinations of receptor complexes can be assembled. Thus, it seems likely that the development of each hippocampal connection depends on the coordination of a particular subset of molecular cues. Below we will discuss the current understanding of the role of semaphorin signaling in the development of each projection.

For the establishment of the main afferent connection, the entorhino-hippocampal pathway (EH), Sema3A, Sema3E, and Sema3F have been implicated [30,83,84,85]. A highly accurate analysis of the mRNA expression of these semaphorins and their receptors has recently appeared in Mata et al., in which it was determined how these molecules undergo fine spatiotemporal regulation in embryonary/perinatal stages [85]. In mouse development, axons from layers II and III of the entorhinal cortex reach the hippocampal white matter and fimbria at E15 and start to invade the hippocampus at E16–E17, reaching the SLM. From E19 onwards, axons cross the fissure and reach the OML of the fascia dentata in its suprapyramidal blade and later, around P2–P5, they invade the infrapyramidal part [86]. According to the literature, axons are forced to move away due to the expression of Sema3A, Sema3E, and Sema3F from the entorhinal cortex and pyramidal and granular layers to reach their proper targets in SLM and OML. Moreover, it has been suggested that low semaphorin expression may form an “axonal corridor” in the subicular region, allowing entorhinal axons to cross this region and reach the hippocampus [85]. However, from the research with Sema3A, Sema3E, and PlexinD1-deficient animals, we may conclude that these molecules are not solely responsible for the proper establishment of the connections. In these animals, the perforant pathway is well developed and only a few axons are misrouted in stratum radiatum and hilus [83,85]. In addition, other guidance molecules such as Ephrin-A3, Netrin-1, and RGMa have been reported to be involved in the layer-specific termination of the perforant pathway in the SLM and OML [39,87,88]. Therefore, the molecules studied to date are not, themselves, essential for this pathway and other guidance factors; a combination of these or different signaling mechanisms should be implicated.

The septal projection, another important input for the hippocampus, originates from cholinergic and GABAergic neurons in the septal region, in particular in the medial septum/diagonal band complex (MSDBC), and their axons arrive in the hippocampus at E17 to finally innervate the stratum radiatum and SLM and the medial portion of the molecular layer (MML) [86]. Furthermore, the hippocampo-septal connection is established earlier on, at E15, which suggests that these axons may serve as a scaffold for the septo-hippocampal fibers. In both of these connections, the actions of Netrin-1 and Sema3A and Sema3F are coordinated in order to form reciprocal connections in both directions of growth [30,83,89,90,91]. In addition, although the participation of Sema3C in the septo-hippocampal connection was suggested by Steup [91], this connection was later reproduced in vitro in the absence of this semaphorin, indicating that its presence is not necessary for its proper development [92]. However, we cannot forget that, as indicated above, these results that contrast those reported in vivo might reflect the limitations of the organotypic slice culture technique when researchers co-culture regions that are not anatomical “neighbours”.

Regarding commissural-associative projections, these fibers originate from the hippocampal pyramidal layer and hilar cells and cross the midline and arrive at the fimbria of the contralateral cortex at E18. After birth, they innervate the stratum oriens, radiatum, and the IML and hilus [86]. To date, it has been suggested that Sema3A-Np1 and Sema3F-Np2 may take part in the process [30,83]. Thus, their high expression in entorhinal axons would prevent commissural axons invading this area and force them to reach the fimbria. Interestingly, only commissural projection, and not entorhinal–hippocampal or septohippocampal projection, is partially mistargeted in *plexin-A3* mutant mice, suggesting that PlexinA3 could be acting as the signaling subunit [93]. Other repulsive cues such as Netrin-1 and Ephrin-A5 expressed in pyramidal and granular cell layers prevent commissural axons from invading these principal cell layers. Moreover, the proper EH connection located in OML may restrict commissural axons to the IML [91,94].

For the guidance of the mossy fibers, the axonal projections of granules cells, it has been reported that Sema3A, Sema3E, and Sema3F participate in their correct targeting [85,95,96,97]. These fibers travel to CA3 via suprapyramidal and infrapyramidal bundles to innervate apical dendrites of CA3 pyramidal neurons in the stratum lucidum. Analysis of *sema3F*-null mice showed an aberrantly-targeted infrapyramidal tract with axons extending into the stratum oriens of CA3 [97]. Also, mice deficient in Sema3F receptors, PlexinA3, or Np2 showed this phenotype [93,96,98,99,100]. However, signaling mediated by Sema3F/Np2/PlexinA3 does not entirely explain the development of the mossy fiber connection because the main bundle (suprapyramidal) normally develops in the absence of this mechanism. Recently, research in mice lacking either Sema3E or its receptor PlexinD1 showed abnormal presence of ectopic mossy fibers and synaptic terminals in the granule cell layer and the IML, although the two principal bundles projected normally [85]. In addition, Nakahara et al. suggested the involvement of Sema3A in mossy fiber innervation though no causal mechanism has been described [95]. Moreover, other guidance molecules distinct from class 3 semaphorins, such as Slit2 and Sema6A, are also implicated. Thus, Slit2 from entorhinal axons in the OML prevent mossy fibers from invading this layer, thus preventing granular axons from innervating their own dendrites [40], while Sema6A and its receptors PlexinA4/PlexinA2 are required for lamina-specific termination [101,102].

Lastly, for the Schaffer-collateral pathway, fibers from CA3 to CA1 pyramidal neurons, little is known about the involvement of guidance molecules. To date, no participation of class 3 semaphorins has been described and only Netrin-1 seems to take part in this process [39].

Taken together, the analysis of research in mutant mice allows us to deduce that axonal pathfinding and lamina-specific innervations in the hippocampus are coordinated spatial–temporal processes and that no single guidance molecule exclusively, but rather a combination of them, may cooperate in regulating these processes. Thus, further research needs to be performed to elucidate what combination of receptors is responsible for the establishment of each pathway.

## 5. Semaphorin-Mediated Signaling Pathways

**Figure 4:**
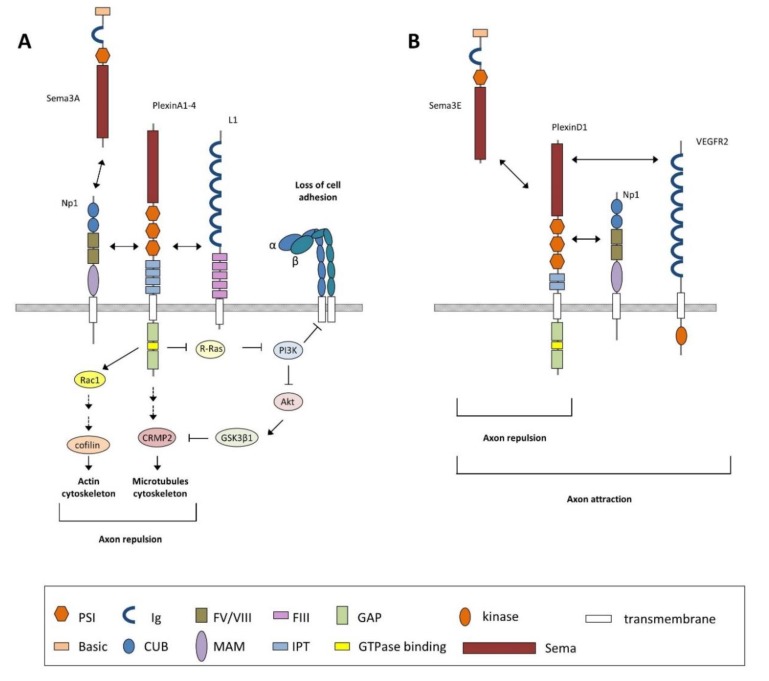
Schematic diagram depicting the main signaling mechanism described for class 3 semaphorins discussed in text (i.e., Sema3A). (**A**) Sema3A-mediated functions are triggered by its interaction with the Np1–PlexinA receptor complex, which promotes the activation of PlexinA through its GAP domain leading to the inactivation of the monomeric G-protein R-Ras and the activation of Rac1. The decrease in active R-Ras downregulates phosphoinositide-3 kinase (PI3K) activity and triggers Akt and GSK3β1 activity, leading to changes in microtubule dynamics. PI3K inactivation also inhibits integrin-mediated cell adhesion to extracellular matrix components. Activation of Rac1 promotes the sequential activation of several GTPases and GTPase-exchange factors (GEFs) such as RhoA, ROCK, and Lim kinases (not shown) to control actin dynamics. Modifications in the cytoskeleton, both in actin and microtubule dynamics, convey decreasing growth cone adhesion, allowing collapse responses. The Np1–PlexinA receptor complex also requires the interaction with Ig-superfamily cell adhesion molecules such as L1 to mediate neuronal functions. (**B**) Unlike other class 3 semaphorins, Sema3E directly binds plexinD1 to induce axon repulsion but induces axon attraction by binding a trimeric receptor complex, namely, Np1-PlexinD1-VEGFR2. Domain abbreviations: CUB = complement binding; FIII = fibronectin type III; FV/VIII = coagulation factor; GAP = GTPase activating protein; Ig = Immunoglobulin-like; IPT = Ig-like Plexin transcription factors; MAM = Meprin, A5, and receptor protein tyrosine phosphatase µ; PSI = plexin-semaphorin-integrin; Sema = semaphorin.

As mentioned above, semaphorins can exert a variety of functions not only in the nervous system but also in other scenarios such as vascular development and tumor progression [62,66], among others. To perform these different roles, semaphorins can bind to different receptors which activate various signal-transducing and modulatory proteins. The list of intracellular molecules involved is continuously growing and it seems that there is no single canonical intracellular pathway. In fact, among one specific class of semaphorins, and also between classes, each member can signal through different divergent pathways involving the participation of different molecules in order to produce different responses [103]. To date, the best-characterized pathway has been the one that mediates axon guidance processes, and which probably governs, with particularities, other neural functions such as dendritic branching, synaptogenesis, and axonal pruning. Despite the fact that these processes imply different subsets of receptors and intracellular molecules, all of them require the reorganization of both the actin and microtubule components of the neuronal cytoskeleton as a final step (Figure 4). In general, all the semaphorin-mediated functions are triggered by the interaction of semaphorin with a member of the plexin receptor family. This leads to the phosphorylation of the plexin cytoplasmatic region and the activation of its GAP domain, which leads to the inactivation of the monomeric G-protein R-Ras. The decrease in active R-Ras downregulates phosphoinositide-3 kinase (PI3K) activity and triggers Akt and GSK3B activity, leading to changes in microtubule dynamics. In parallel, microtubule dynamics is also modulated by the participation of Cdk5 after Sema3A and Np1/PlexinD1 interaction [104]. In fact, both GSK3β and Cdk5 activities triggered by secreted semaphorins have been reported in healthy development and during neurodegeneration (see below). Apart from R-Ras, other GTPases and GTPase-exchange factors (GEFs) such as RhoA (Ras homolog gene family, member A), ROCK (Rho-associated, coiled-coil containing protein kinase), and Lim kinases participate in the process to finally regulate actin dynamics. These modifications in the cytoskeleton, both in actin and microtubule dynamics, convey decreasing growth cone adhesion, allowing collapse responses. Apart from plexins, class 3 semaphorins, except Sema3E, require neuropilins (Np1 and Np2) as ligand-binding co-receptors to signal through class A plexins. In addition, other molecules have been seen to be involved as either semaphorin receptors or components of a holoreceptor complex: Otk (off-track), L1 IgCAM, Met, CD72, Tim-2, heparan and chondroitin sulfate proteoglycans (HSPGs and CSPGs), and integrins [55,103,105]. Interestingly, it has been reported that certain transmembrane semaphorins (class 1, 4, 5, 6) can also act as receptors, a process which is called bi-directional signaling [106,107]. Furthermore, an exciting feature of Sema3E was described in 2007, reflecting the high versatility of these molecules. In this case, the authors identified Sema3E as a repellent for both corticofugal and striatonigral connections by its interaction with PlexinD1. However, when Sema3E binds to the PlexinD1/Np1/VEGFR2 receptor complex, the final response obtained was axonal attraction for the subiculo-mammilary tract. This fact suggests that Np1 can exert a “gating” function by reversing the response triggered by PlexinD1 [108]. Thus, the great ability of semaphorins to bind to different receptors permits multiple combinations of receptor complexes to be assembled, and therefore a large variety of signaling pathways can be used and cellular responses carried out.

## 6. Involvement of Semaphorins in Neuronal Disorders

Growing evidence suggests that semaphorins, their receptors, and also their intracellular signaling components are involved in epilepsy and some neurodegenerative disorders. Epilepsy includes a variety of different neurological disorders characterized by the predisposition to recurrent unpredictable seizures caused by disturbances in the electrical activity of the brain. These failures come from abnormal synaptic connections originating during brain development but also triggered by different adult pathological conditions [109]. The most frequent focal epilepsy in adults is the mesial temporal lobe epilepsy (MTLE) characterized by hippocampal sclerosis, cell loss in the hilus and CA1, ectopic granule cells, and abnormal mossy fibers sprouting into the IML of the dentate gyrus [110]. Traditionally, it was accepted that mossy fiber sprouting contributes robustly to the hyperexcitability of the hippocampus, but this pro-epileptogenic role has been questioned recently by Cavarsan et al. because its presence is not necessary to develop MLTE. Despite the controversy concerning whether aberrant sprouting may be a cause or a consequence of the seizures, it seems likely that the rearrangement of neuronal circuitry observed in epileptic hippocampi implies the participation of certain guidance molecules in the process [111]. In 2003, Holtmaat et al. observed that after induction of status epilepticus, Sema3A was temporarily downregulated in the entorhinal cortex, the main afferent of dentate gyrus, prior to the appearance of mossy fiber sprouting, suggesting that its downregulation could facilitate the formation of recurrent projections of mossy fibers after seizures [112]. Furthermore, it has been reported that Sema3A is elevated in cerebella of schizophrenic subjects and appears to contribute to the synaptic pathology of schizophrenia [113], and it is also increased in hippocampi in a murine model for this disease [114]. Barnes et al. described a reduction of Sema3C and Sema3F mRNAs in CA1 pyramidal cells and also a reduction of Sema3F and Sema4C gene expression in the CA3 region after kainic acid-induced status epilepticus, suggesting that these changes could be related to aberrant synaptogenesis observed in both of these areas [115]. As we mentioned above, Sema3F acts preferentially through the Np2/PlexinA3 holoreceptor complex to develop its function. Mice deficient in any of the components of this complex exhibit the same abnormal phenotype regarding mossy fiber projection, which indicates that this signaling is important not only for appropriate development but also for the correct excitatory synaptic transmission of this connection. Thus, several studies have reported that mice lacking Sema3F or Np2 were prone to seizures [96,98,116,117], and presented impairments in both hippocampal-dependent memory tasks and motor behavior [118] or abnormal anxiety-related behavior [119]. In addition, the application of secreted Sema3F to acute hippocampal slices modulated both the frequency and amplitude of EPSCs (excitatory postsynaptic currents) in granule cells and pyramidal neurons [116]. Furthermore, mossy fiber sprouting, in parallel with a decrease in Sema3F gene expression in dentate gyrus, was recently described in another model of epilepsy, the lithium–pilocarpine-induced status epilecticus model, suggesting a close association between these events [120]. Moreover, as we mentioned above, Sema3E-deficient mice showed abnormal presence of ectopic mossy fibers and synaptic terminals in the granule cell layer and in the IML, as well as enhanced neural excitability in the dentate gyrus, although they did not present epileptic-like activity [85]. In line with this, it was reported that these mice also presented reduced anxiety levels and moderately impaired spatial working memory [108], suggesting that their aberrant phenotype is related to altered excitability and an epileptic-like pattern. Further studies are needed to determine whether dysregulation in semaphorins and/or their receptors’ expression contributes or causes epilepsy phenomena.

Among neurodegenerative disorders, evidence suggests a close relation between semaphorins and their receptors in Alzheimer’s disease (AD) and amyotrophic lateral sclerosis (ALS). AD is, in a broad sense, characterized by the presence of progressive neuronal degeneration accompanied by relevant neuroinflammation [121]. The principal histopathological hallmarks of AD are the presence of neuritic and cerebrovascular plaques containing β-amyloid (Aβ) peptides and intraneuronal neurofibrillary tangles enriched in hyperphosphorylated tau protein, and the degeneration of hippocampal CA1 and subicular pyramidal neurons [122]. In 2004, a multiprotein complex from the hippocampus of patients with AD was isolated and characterized as containing phosphorylated MAP1B, collapsin-response mediator protein 2 (CRMP-2), Plexins A1 and A2, and a processed form of Sema3A [123]. In adult rodent hippocampal formation, Sema3A is expressed by EH-projecting cells as well as by subicular neurons [124]. Early stages of AD are characterized by the death of the projecting entorhinal neurons as well as changes in intrinsic hippocampal connections (see Figure 7.3 in [125]). Thus, due to these neurodegenerative-mediated changes, it was proposed that the aberrant release of Sema3A from subiculum during early clinical stages of AD resulted in the internalization and transport of Sema3A to CA1, and that this may contribute to the degeneration of neurons in the CA1 field and therefore the decrease of hippocampal functions observed in the disease [123]. In addition, CRMP-2 is an essential component of Sema3A-induced growth-cone collapse and, interestingly, phosphorylated CRMP-2 was found in neurofibrillary tangles in brains of autopsied AD patients [126]. In addition, it is phosphorylated by Aβ through a RhoA GTPase-dependent mechanism in an AD animal model [127]. Moreover, it was observed that the sequential phosphorylation of CRMP-2 by Cdk5 and GSK3B is an important process of Sema3A signaling, and that the same mechanism is involved in the pathological aggregation of microtubule-associated proteins [128]. However, it needs to be pointed out that an association study for single nucleotide polymorphisms for Sema3A and Sema4D was carried out in an Italian population resulting in no direct correlation between these polymorphisms and the susceptibility to develop AD [129]. Furthermore, in an attempt to discover new mechanisms that could explain the genetic contribution to AD, Jun et al. performed a family-based genome-wide association study in which they identified PlexinA4, a receptor for secreted Sema3A and class 6 (Sema6), as a risk factor in AD pathogenesis or progression by contributing to the acceleration of tau phosphorylation, leading to neurofibrillary tangle formation [130]. The evidence suggests a cross-talk between Sema3A, Aβ, and cytoskeleton rearrangement signaling mechanisms that needs to be analyzed in depth. The role of other semaphorins and/or receptors in AD has not been greatly explored and few data have been published to date. Thus, a decrease in Sema3F (formerly called H.SemaIV) in the cerebral cortex and the hippocampal formation was described in AD patients but it was also abnormally accumulated in specific neuronal compartments such as perikarya and fibers. These findings do not seem to be directly related to the pathogenesis of AD but are probably due to neuronal loss and altered axonal transport in degenerating neurons [131]. Lastly, a neuroprotective role for Sema3C in AD was proposed as a result of the observation that upper layers of EC presented a decrease in Sema3C mRNA content with respect to lower layers in individuals with no clinical evidence of neurological disease, which may explain why upper neurons are more vulnerable to the onset of AD than those located in lower layers [132]. Interestingly, a recent genome-wide association study to analyze genetic risk factors for posterior cortical atrophy, a rare variant of AD, proposed three candidate loci, *SEMA3C*, *CNTNAP5*, and *FAM46A*, as potential genes of interest in this disease [133]. As indicated, neuroinflammation and microglial activation play crucial roles during AD progression (see above references and [134]). Very few data have been published analyzing the participation of semaphorins and their receptors in modulating neuroinflammation in AD. However, parallel studies using lipopolysaccharide (LPS) treatments in vitro and in vivo leading to microglia activation demonstrated that PlexinA1 and Np1 are over-expressed by activated microglial cells [135] and early AD stages are characterized by a burst of Sema3A expression by hippocampal neurons [123]. This Sema3A might trigger microglia cell death, as described in other neuroinflammatory models, reducing their phagocytic-promoting functions which are necessary to reduce amyloid burden in affected brains [136]. Moreover, pioneer studies demonstrated a decrease of synaptic contacts generated by fast-spiking Parvalbumin (PARV)-positive interneurons in the hippocampus of AD patients and mice models [137,138,139]. In a recent study, using cutting-edge optogenetic techniques, Iaccarino et al., increased PARV-activity in a mouse model of AD (5 × FAD mice), leading to increased microglia activation and reducing amyloid burden in treated mice [140]. Taken together, we can hypothesize that decreased inhibition associated with PARV-positive neurons in AD might also contribute to the increased Sema3A in affected AD patients.

Altered semaphorin function or expression is not confined exclusively to degenerative diseases related to the hippocampus. In fact, it is also well known that there is an association of certain semaphorins with other diseases, such as Sema4A with retinal degeneration [141], Sema3A-Np1 signaling with motor neuron degeneration [142,143], Sema3A, Sema7A, and Sema4D with multiple sclerosis [144,145], and Sema3A and Sema5A with Parkinson’s disease [146,147] (see also [47] for review).

In addition, semaphorin signaling has also been implicated in the pathogenesis of many congenital diseases (see [148] for review); its relation with Kallmann syndrome (KS) is one of the most analyzed. KS is a genetic disease characterized by hypogonadotropic hypogonadism and an impaired sense of smell and is thought to be due to a developmental defect in the migration of gonadotropin-releasing hormone (GnRH) neurons. Previous studies identified altered Sema3A/Np1/Np2 signaling in the pathogenesis of the disease, with normal function being necessary for the proper migration of GnRH into the brain [149,150]. However, recently, another semaphorin, Sema3E, due to its interaction with PlexinD1/VEGFR2, has been proposed as being essential for GnRH neuron survival [151]. Thus, both Sema3A and Sema3E may be implicated in the same disease but playing different roles: mutations in Sema3A would cause the ectopic migration of GnRH neurons and Sema3E would act as a survival factor for the neuron population by triggering anti-apoptotic mechanisms.
